# Does Pictorial Composition Guide the Eye? Investigating Four Centuries of Last Supper Pictures

**DOI:** 10.16910/jemr.13.2.7

**Published:** 2020-08-21

**Authors:** Rosa Sancarlo, Zoya Dare, Jozsef Arato, Raphael Rosenberg

**Affiliations:** University of Vienna, Austria; Leuphana University, Lüneburg, Germany

**Keywords:** Eye movement, eye tracking, composition, saccades, art perception

## Abstract

Within art literature, there is a centuries-old assumption that the eye follows the lines set out by the composition of a painting. However, recent empirical findings suggest that this may not be true. This study investigates beholders’ saccadic eye movements while looking at fourteen paintings representing the scene of the Last Supper, and their perception of the compositions of those paintings. The experiment included three parts: 1) recording the eye movements of the participants looking at the paintings; 2) asking participants to draw the composition of the paintings; and 3) asking them to rate the amount of depth in the paintings. We developed a novel coefficient of similarity in order to quantify 1) the similarity between the saccades of different observers; 2) the similarity between the compositional drawings of different observers; and 3) the similarity between saccades and compositional drawings. For all of the tested paintings, we found a high, above-chance similarity between the saccades and between the compositional drawings. Additionally, for most of the paintings, we also found a high, above-chance similarity between compositional lines and saccades, both on a collective and on an individual level. Ultimately, our findings suggest that composition does influence visual perception.

## Introduction

Art literature explains the features of art and architecture by describing the movement of the eye as early as the 6th century AD. From the 18th century on, the composition of paintings is explicitly described in terms of lines that lead the eye of the beholder through the artwork. In “The Analysis of Beauty,” Hogarth (9, p. 25) suggested that the eye follows “weaving and serpentine lines” which are constructed by the forms and objects in a painting. A decade later, Denis Diderot advanced this argument, saying that every painting must have a well-constructed “line of liaison” that “will serve as a guide to anyone looking at [the picture] as well as to anyone attempting to describe it” (5, p. 152). Such descriptions of the viewer’s eye moving along abstract lines through the composition of a painting became frequent in 20th century literature (8; 14; 20; 10; 1; 13; 19; 2). Since the 18th century, the drawing of such composition lines has also become more and more common in the context of art education ( 17). While the planimetric notion of composition is rather recent, Thomas Puttfarken (15) argues that it has been an active principle of Western painting since just before the Italian Renaissance, even though it was not written about as such at the time.

In 1935, Thomas Buswell wrote the first book about eye tracking in regard to pictures. He demonstrated that the eye does not follow a smooth line through a painting but instead jumps rapidly without a semblance of linear progression among points of interest. Despite the sporadic movements of the eye and differences among participants, however, he found that every painting had points that attracted a higher number of fixations for all participants. He also found that the direction of eye movement generally follows the “principal lines in a picture.” By this, Buswell was referring to vertical and horizontal lines prominent in the elements constructing a scene (3, p. 82). Later, Alfred Yarbus (21) further demonstrated that not only do gaze movements differ among participants, but also differ according to the tasks they were given. When asked to simply view the painting The Unexpected Return by Ilya Repin the gaze path of one participant was significantly different than when this same person was asked to assess the ages of the figures in the painting or the material situation of the family depicted. Yarbus, however, noted that there is a “cyclical pattern” in how participants viewed Repin’s painting, returning again and again to the same points of interest (21, p. 194). These studies laid the foundation for the empirical study of artworks with eye tracking devices. At the onset, both Buswell and Yarbus dismissed the notion of a single continuous gaze path that followed the composition of a painting. They noticed however that eye movements do follow patterns given by the key elements of a picture, but they did not study the nature of those patterns.

In recent years, there has been a resurgence of interest in eye tracking studies that test the effects of composition. Garbutt & Spehar (7) and Kirtley (11) have focused on the lines of composition explicitly described by artists or art historians and concluded that the eye does not follow a sequential and linear path through paintings. They focused their analysis on (in Garbutt’s case), fixations alone, or (in Kirtley’s) the entire sequential, linear scan path. Thus far, little attention has been paid to the findings Buswell and Yarbus reported, which seemed to support the art historical literature—namely the repetition of patterns of a participant’s gaze and viewing direction that might be consistent with the compositional lines of a painting. Moreover, all of the recent studies claim to be preliminary, a proof of concept for the effects of eye tracking in studies of art, or a pilot study to encourage further research using these methods. By taking so literally the sequential progression of the eye discussed in art literature, they focus on what the eye does not do—sequentially follow the line of the composition—rather than on what the eye does, and how composition might play a role in influencing this.

Recent studies at the Lab for Cognitive Research in Art History (CReA) at the University of Vienna have demonstrated that composition may, after all, have an influence on eye movement (18): though the eye does not follow a line sequentially, compositional lines do emerge in the patterns of repeated saccades between key elements of a painting. These studies used a different method of analysis, looking at saccades instead of fixations, and focusing on cumulative saccades instead of sequential gaze paths. They therefore developed innovative tools for the visualization of saccade patterns (12). However, they did not study the relation between composition and saccadic eye movements experimentally and could not provide a quantitative method for such a comparison. Therefore, in the current study we aim to test these preliminary findings. We compare two different levels of perception of the same group of participants, namely, 1) visual perception, by using an eye tracker; and afterwards, 2) cognitive perception of composition by means of a drawing task. We analyzed the similarity of the data between the participants on both levels and their similarity between the levels, i.e. between saccades and drawn composition lines.

We hypothesized that: 1) there is a high degree of similarity between the composition lines drawn by participants for the same painting; 2) there is a high degree of similarity between the saccades of the participants looking at the same painting; 3) there is a degree of similarity between drawn compositional lines and saccades made while looking at the same painting; 4) the representation of space influences both the perception of painted compositions, and in a similar way, the saccades of the beholders.

We investigated these hypotheses with an experiment consisting of three consecutive tasks: 1) a viewing task, during which participants were asked to view fourteen paintings representing the Last Supper from the 12th to the 16th century, while their gazes were recorded by an eye tracker; b) a composition drawing task, during which they were asked to draw the main lines of the composition of the same paintings; and c), a space rating task, where the participants were asked to rank reproductions of the same paintings based on depth. We chose the biblical scene of the Last Supper since it is a very common motif in the history of Western painting. There are many examples over several centuries with a wide range of compositional strategies and pictorial styles, spanning from the rather flat plane surface representation of the Middle Ages to the perspectival constructions of Renaissance art and the complex diagonal spaces of Mannerism.

## Methods

### Participants

Participants in the experiment were recruited among art history (major) undergraduate students at the University of Vienna. Thus, they were familiar with the concept of composition, but were not yet experts in the field. All were naive to the purpose of the study and were paid €10 for their participation. The sample consisted of forty participants. All had normal or corrected-to-normal vision and no dyschromatopsia as assessed by Ishihara color plates. Eight participants were excluded due to insufficient recording quality. The data of the remaining thirty-twos were included in the analysis (age range 19–51, mean 25.7). In order to exclude effects due to gender we only recruited female participants.

### Materials

The stimuli consisted of reproductions of fourteen paintings representing the Last Supper from the 12th to 16th century (See Fig. A for a list of the stimuli. See in the appendix Figs. A1 to A14 for the reproductions of the paintings):

Fig. A. List of Stimuli:

Nicholas Von Verdun, The Last Supper from the Verdun Altar, 1181, enamel, Leopold Chapel of the Monastery of Klosterneuburg, Austria

Giotto di Bondone, The Last Supper, 1306, fresco, Scrovegni Chapel, Padua, Italy

Pietro Lorenzetti, The Last Supper, 1320, fresco, San Francesco Lower Church, Assisi, Italy

Andrea del Castagno, The Last Supper, 1445-50, fresco, Saint Apollonia, Florence, Italy

Dieric Bouts, The Last Supper from the Altarpiece of the Holy Sacrament, 1465, oil on panel, St. Pieterskerk, Louvain, Belgium

Domenico Ghirlandaio, The Last Supper, 1480, fresco, Ognissanti Monastery Refectory, Florence, Italy

Luca Signorelli, Communion of the Apostles (The Last Supper), 1512, oil on panel, Diocesan Museum, Cortona, Italy

Unknown Netherlandish Painter, The Last Supper, Central Panel of Triptych, 1515-1520, oil on wood, The Metropolitan Museum of Art, New York City, USA

Lucas Cranach the Elder, The Last Supper, central panel of the Reformation Altarpiece, 1547, oil on panel, St. Mary Protestant Church, Wittenberg, Germany

Juan de Juanes, The Last Supper, 1555-1562, oil on panel, Prado Museum, Madrid, Spain

Jacopo Tintoretto, The Last Supper, 1578, oil on canvas, Scuola Grande di San Rocco, Venice, Italy

Paolo Veronese, The Last Supper, 1585, oil on canvas, Pinacoteca of Brera, Milan, Italy

Jacopo Tintoretto, The Last Supper, 1592, oil on canvas, Basilica of San Giorgio Maggiore, Venice, Italy

Federico Barocci, The Last Supper, 1608, oil on canvas, Cathedral of Urbino, Italy

We used high-resolution reproductions and presented them on a 3840 x 2160 pixel BENQ LCD monitor, using a maximum of 2880 pixel width (in order to minimize the distance between participants and monitor) and corresponding height, so that the original proportions of the pictures were preserved. The movements of the dominant eye of each participant over the stimuli was recorded using the EyeLink 1000 Plus remote eye-tracker at a 1000 Hz monocular frequency. The drawing task was performed on an iPad and the rating task was done with cards printed with reproductions of the fourteen paintings.

### Design

The purpose of the study was not mentioned before or during the experiment, in order to avoid influencing the participants’ actions. Participants were informed that the study was about the Last Supper, duration and structure, and that all data would be collected anonymously. They were asked for written consent in accordance with the Declaration of Helsinki and the University of Vienna’s own regulations. Participants then completed a vision and color blindness test. The dominant eye was determined and used for recording. The experiment consisted of three consecutive tasks: viewing, composition drawing, and rating space.

1. Viewing: Participants were shown the fourteen paintings in a random order for sixty seconds each, while we registered their eye movements. Seated one meter from the screen, they were asked to view the paintings as if they were in a museum and to rate each painting immediately after it was shown on a Likert scale (from 1 = I like it very much, to 5 = I do not like it at all). The answers to these questions were not analyzed. The aim was to facilitate a free, aesthetic-oriented viewing (that is, with no purpose other than enjoyment of the picture). The presentation of each painting was preceded by a black screen with a calibration point to check, and if necessary correct, the calibration of the eye tracker as well as to make sure that each participant would start viewing the image from the same position.

2. Drawing Composition: The participants were shown the fourteen paintings once again on a tablet, and asked to finger-draw each work’s composition on a tablet. The instruction for this task was given as follows: “Art Historians tend to define composition as the most important lines for the structure of the painting. Please draw the lines that, in your opinion, are the most important for the composition of the following painting.”

3) Rating Space: The participants were given prints of the fourteen paintings and asked to order them according to the perceived degree of depth. In order to quantify this order, we assigned a score to each painting from 1 for the flattest and up to 14 for the one representing the greatest depth. The instruction for this task was given as follows: “You will now be given the reproductions of all the images you have already seen. Please order them on the table according to the amount of depth represented: from the most flat to the one with the most depth.”

### Data Analysis

To analyze eye tracking data we used the proprietary SR Research acceleration and velocity algorithm to detect fixations and saccades. We focused the analysis on saccades, independently from their sequence and moment of time within the sixty seconds of viewing.

To analyze compositional drawings we overlaid all of the lines drawn by every participant for each painting. The visualizations of all saccades of all participants and of all lines drawn by all participants (see Appendix Fig. A1 to A14) show a great overlap, hence a high amount of repetition. Also there seem to be evident similarities between saccades and drawings in regard to most paintings. In order to quantify such comparisons, we defined a similarity index that allowed for the calculation of the similarity/diversity of saccades and compositional drawings between the participants, as well as the similarity of saccades and drawings to each other.

**Figure 1.**
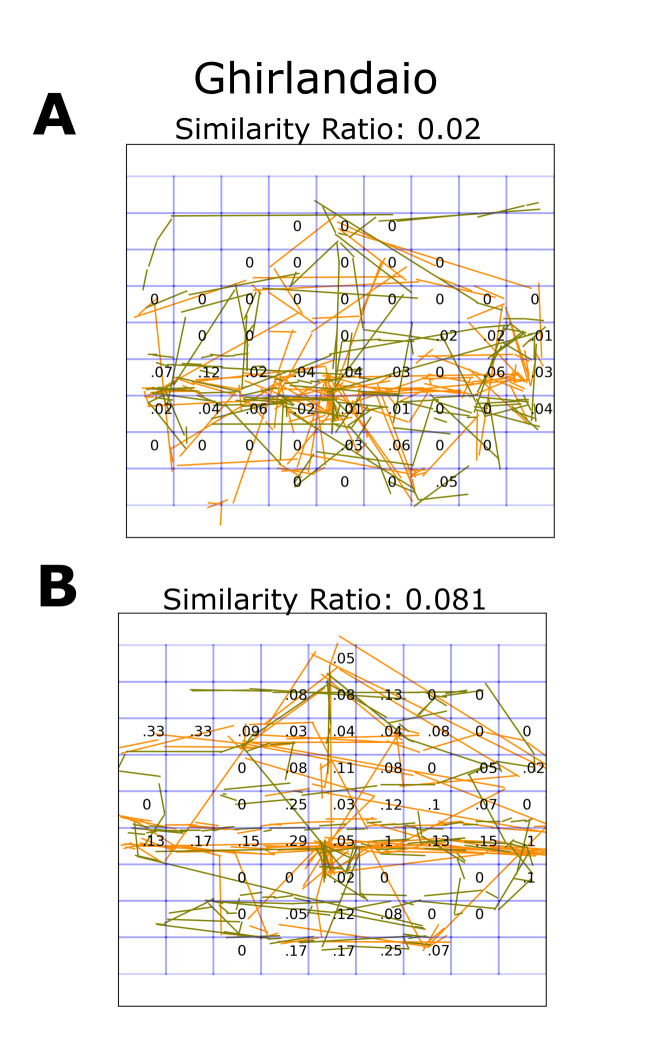
Data Analysis example. A relatively dissimilar (top) and a highly similar (bottom) pair of observers’ saccades during one minute of free viewing are visualized for the same painting (Ghirlandaio). The saccades of each participant are traced in different colors (yellow and green) The blue grid shows the spatial resolution of the calculation (9*9). Each cell shows the similarity ratio of saccadic angles (number of similar pairs of saccades using a threshold of 5° divided by the number of comparisons). The average similarity ratio is shown in the title. The only cells that were analyzed were those in which both participants had crossed cell boundary E.

**Figure 2.**
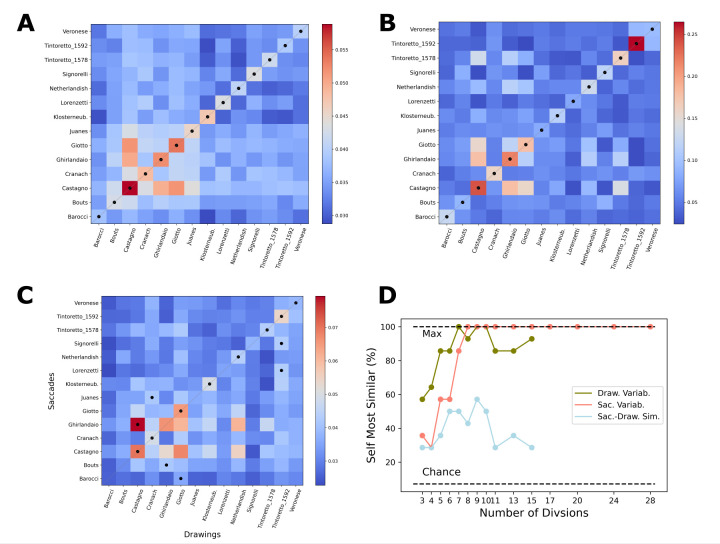
Comparison across paintings as method validation. The similarity across different paintings is shown on A-C, with black dots marking the most similar painting for each row (red = higher similarity). A) Saccade Variability. Eg: the 14 cells in the first row represent similarity between all saccades of the Veronese painting compared to all of the saccades for each of the 14 paintings (across different observers). B) Drawing Variability, the same as A but for drawings. C) Saccade-Drawing Similarity (xaxis: drawings, y-axis: saccades). Cells in the first row show the similarity of all saccades for the Veronese compared to the compositional drawings of all 14 paintings. D) This figure shows the combined number of self-similar paintings (number of black dots falling on diagonal in A-C) for the 3 measures for the different number of divisions (x-axis), where the most similar set of saccades/drawings are the ones for the same paintings.

### Analysis of Saccades

The paintings were divided into an n x n grid (n={3,4,....,10,11,13,15,17,20,24,28}) (blue grid in Fig. 1). For each division, we extracted the angle of all saccades in every cell they crossed (thereby ignoring very short saccades). This saccadic angle was compared for every cell across all pairs of different participants looking at the same paintings and for all combinations of paintings. We used 5° as the threshold. Hence, all angles differing less than 5° were counted as similar. We thereby did not take into account the direction of the saccade; hence, for example, 185° was counted as similar to 3°. We then counted the number of similar saccades and divided it by the total number of comparisons in a cell. This resulted in a similarity ratio for each cell. Finally, the similarity ratio was first averaged across cells, then across participants, to obtain an overall measure of saccade similarity for all combinations of paintings. To summarize, the similarity ratio (SR) of two participants (s1,s2) for a given painting was calculated as:

**(1):**
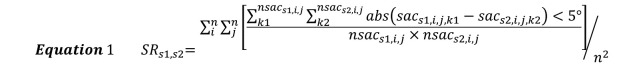


In the above equation, i is horizontal, j is the vertical cell number, n the number of cell divisions, nsac_s,i,j_ is the number of saccades in a cell, sac is the saccadic angle of the k_th_ saccade.

**Figure 3.**
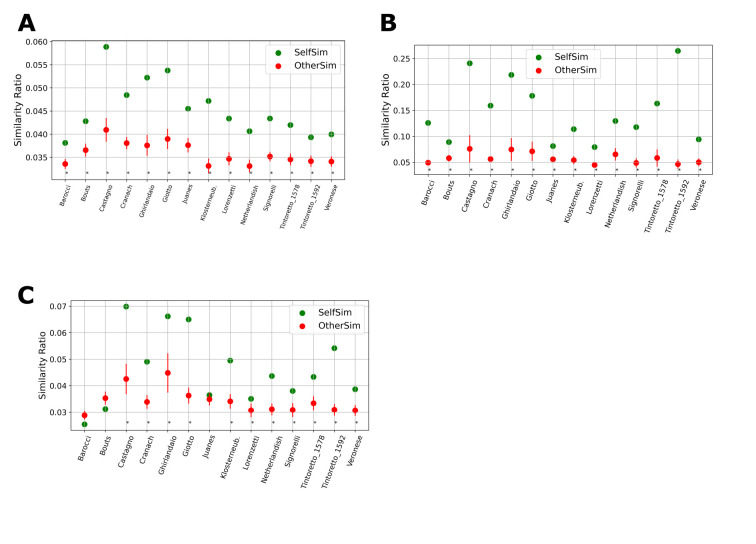
All measures are sensitive. A) Saccade Variability: for each painting the similarity ratio of saccades to saccades from the same painting is shown in green and is always significantly above the average similarity from other paintings (red). B) Drawing Variability: the similarity ratio of drawings to drawings from the same painting (green) is significantly above the average similarity to drawings from other paintings (red). C) Saccade-Drawing Similarity: the similarity ratio of saccades to drawings from the same painting (green) for most paintings is significantly above the similarity ratio comparing those saccades to drawings from other paintings (red), thereby demonstrating a relationship between the shape of the saccades and the drawn composition lines. These figures are based on 9*9 divisions grid. (Error bars: SEM, * p<.01, one-sample t-test)

In order to validate this method and find the number of divisions necessary for a reliable analysis, we tested if saccades of any given participant viewing one painting are more similar to the saccades of another participant looking at that painting or the saccades occurring over other paintings. The comparison was run with any number of cells from 3*3 to 28*28. We found that if the number of cells is at least 8*8, all of the fourteen paintings are self-similar (Fig. 2D). This was true for all divisions above 8*8 as well, demonstrating both a stimulus-specific influence on saccade patterns and the validity of our method as long as cells are not too large. To test if differences in similarity are significant, for each division and painting we contrasted the similarity value obtained for a painting to the similarity values obtained by comparing that painting to each of the thirteen other paintings by a one-sample t-test (self vs. other similarity). Fig. 3A illustrates the degree of similarity to the saccades performed while looking at the same and other paintings with a grid of 9*9 divisions.

### Analysis of Composition Lines

For the comparison with the drawn lines of composition, we used the same grid-based approach as outlined above for the saccades with n horizontal and n vertical cell divisions (n={3,4,…10,11,13,15}). We did not use as fine-grained divisions as with saccades, due to the lower spatial accuracy of the lines drawn with fingers on the tablet. Whereas saccades could be abstracted as straight lines conjoining two points (i.e. fixations), the composition lines drawn by participants often deviated—more or less voluntarily—from a straight line course. Therefore, in the course of pre-processing, the drawn composition lines were segmented at acute angles, and approximated with straight lines within the cells of the grid. Then, the angle of the approximated lines was calculated for each cell. Based on these angles, we calculated the similarity of composition lines across participants (as was described above for saccades), using for saccades a similarity threshold of 5°. The similarity ratio for participants s1,s2 was calculated as:

**(2):**
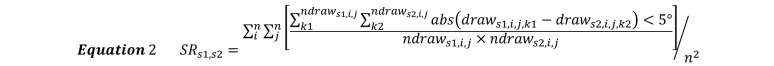


In the above equation, i is horizontal, j is the vertical cell number, n the number of cell divisions, ndraw_s,i,j_ is the number of drawing lines in a cell, draw is the angle of the k_th_ drawing line.

Comparison of Saccades and Composition The similarity between composition lines and saccades was calculated across all participants and paintings, using the same procedure, the same divisions (n={3,4,…10,11,13,15}), and the same threshold (5°) as described above. The similarity ratio of participants s1,s2 was calculated as:

**(3):**
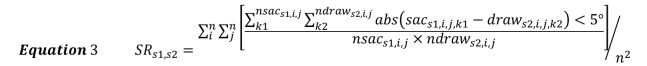


In this equation, i is horizontal, j is the vertical cell number, n the number of cell divisions, ndraw_s,i,j_ is the number of drawing lines in a cell, nsac_s,i,j_ is the number of saccades in a cell, sac is the saccadic angle of the k_th_ saccade, draw is the angle of the k_th_ drawing line.

### Analysis of Space Ratings

In order to analyze space ratings we added the single ranking that every participant gave to each painting (one to fourteen). The accumulated scores served to rank the degree of depth for each painting.

## Results

For each painting we quantified: 1) the similarity between the saccades of different participants; 2) the similarity between the compositional drawings of different participants; 3) the similarity between saccades and compositional drawings for each participant; and 4) across all participants. The analysis was conducted using fifteen different grid divisions for each painting, which allowed more robust statistical comparisons than analyzing only a single division. However, in order to visualize the data, the 9*9 division is used on all figures, as it was highly-self similar relative to “other similarity” (Fig. 2).

### Similarity of cumulative saccades

Using grids of at least 6*6 cells, the saccades of all fourteen paintings were significantly (t(13), *p*<.01 for all fourteen comparisons) more similar to saccades from the same painting than to saccades from different paintings. Furthermore, if the grid was divided into 8*8 cells or higher, the most similar set of saccades, for all fourteen paintings, were the saccades from the same painting (chance=1, Fig. 2D).

**Figure 4.**
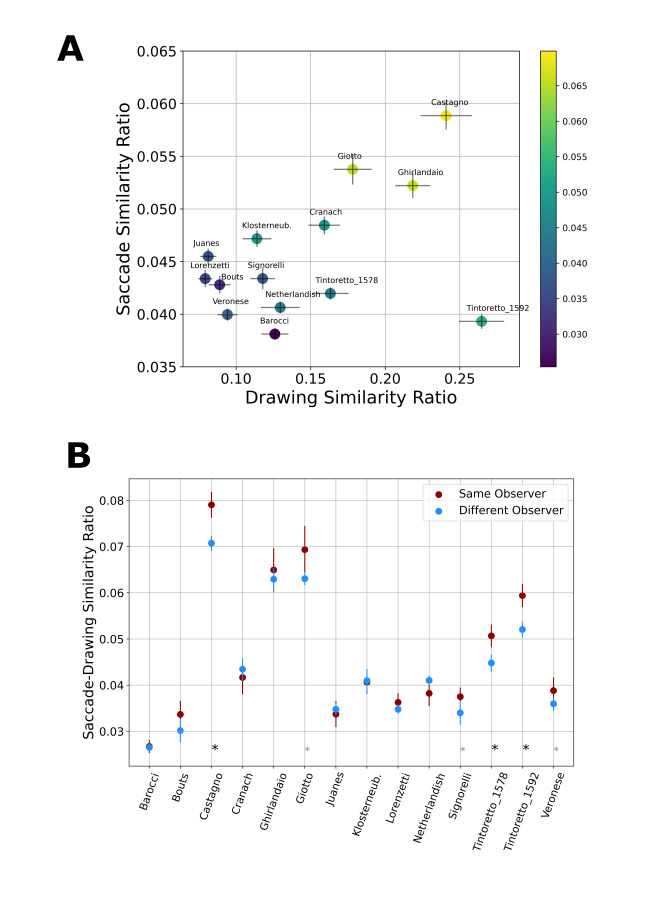
Similarity Measures by Painting. A) Drawing Variability (x-axis) and Saccade Variability (y-axis) only showed a weak correlation, but both measures are highly correlated with Drawing-Saccade similarity (yellow-highly similar, blue: not similar) (Error Bars: SEM). B) Saccade-Drawing similarity was higher for within observers (blue line) than across different observers (brown line) ( *p <.05, *p<.01, Error Bars: SEM across divisions).

Analyzing the relative similarity of saccades in different paintings across the different divisions (using divisions 8*8 and above) showed that paintings differ reliably: for some paintings cumulative saccades are more similar, for other paintings they are more different, i.e., they elicit more diverse saccades (Fig 3A, Fig 4A). The paintings eliciting the most uniform saccades were those by Castagno, Giotto and Ghirlandaio, with a similarity score that was significantly higher than all of the other paintings (*p*<.001 for all 33 comparisons between paintings). The painting by Barocci stimulated the most variable saccades, with similarity scores significantly below all other paintings (*p*<.001 for all 13 comparisons with the other paintings). We also compared the similarity of saccades within participants (for each painting) with the similarity of saccades between participants (for each painting). Interestingly these measures are highly correlated across paintings (*r*=.912, *p*<.001). “

### Similarity of cumulative compositional drawings

Results of the similarity algorithm show that compositional drawings for any of the paintings were more similar to drawings of the same painting than to the average similarity of drawings for any of the other paintings (Fig 3B, all comparisons significant, within all divisions). Furthermore, by using anything between 7*7 and 10*10 divisions, all of the paintings were self-similar: the most similar set of drawings to each drawing were the ones for the same painting (Fig 2D). The self-similarity coefficients drops slightly for divisions above 11*11, reflecting the limited spatial accuracy of the compositional drawings made on the tablet by the participants with their fingers. The painting with the least variable drawings was the 1592 Tintoretto (Fig 3B, Fig 4A). It was followed by those of Castagno, Giotto and Ghirlandaio, which also had relatively similar saccades.

### Similarity of cumulative saccades and cumulative compositional drawing

When comparing cumulative saccades and cumulative compositional drawings, the results of the similarity algorithm show that, depending on the number of divisions, for most of the paintings the saccades of participants looking at a painting were more similar to the compositional drawings made for that same painting (Fig 3C). For grid divisions of 8*8, 9*9 and 10*10, the average similarity of drawings and saccades of the same painting was significantly higher than for any other paintings, except for these three out of the fourteen paintings: those by Barocci, Bouts, and Juanes. The number of paintings, where (compared to all saccades) the most similar composition lines were those drawn for the same painting, varied, depending on the number of divisions (Fig 2D). This “self-similarity” number was the highest, eight (chance=1), using the 9*9 division. Like the drawing similarity measures, the self-similarity scores drops abruptly after 11*11 divisions due to the limited spatial accuracy of the drawings.

Overall, as with saccade to saccade comparisons, the works by Castagno, Giotto and Ghirlandaio had the highest, and Barocci the lowest, similarity ratios in the comparison of saccades and drawings (Fig 3C, Fig 4A). The similarities between Castagno, Giotto and Ghirlandaio were significantly above, and the similarity of Barocci was below all other paintings (*p*<.001, all 46 comparisons).

### Individual similarity of saccades and compositional drawings

We also measured the similarity between compositional drawings and saccades on an individual level and for every painting. We then compared this similarity across the participants of the experiment. In order to test if saccades from a participant were more similar to her drawings, for each painting, we compared the similarity of drawings and saccades within observers to the similarity of drawings and saccades across different observers (using all divisions between three and fifteen). We found that the overall similarity of drawings and saccades is higher within a single participant than across different participants (Fig. 4B, *t*(13)=2.7107 *p*=.0178). Analyzing this difference separately for each painting, we found that the difference in similarity within the same observer as opposed to across different observers is the largest for Castagno, Tintoretto 1578, Tintoretto 1592 ( *p*<.01), followed by Giotto, Signorelli and Veronese ( *p*<.05).

### Relationships between measures

There is no significant correlation between saccade variability and drawing variability across the paintings (Fig4A, *r*=0.4395, *p*=.1159 *r*_s_=0.22, *p*=.4456). However, both measures are highly correlated with the saccade-drawing similarity (Saccade variability and Saccade-drawing similarity: *r*= 0.81, *p*<.001, Drawing variability and Saccade-drawing sim: *r*=0.79, *p*<.001). This strong relationship is further supported by Spearman correlation (*r*_s_=0.6 *p*= 0.0233, *r*_s_=0.789 *p*<.001), excluding the possibility that outliers are responsible for this relationship. In a combined model, using cross-validated Lasso regression, we estimated the relative importance of these two measures on saccade-drawing similarity. This analysis showed that these measures have a very similar effect (ꞵ _saccade_=.0068; ꞵ_drawing_=.0072) in predicting saccade-drawing similarity, but note that these results must be interpreted with caution due to the limited sample size (N=14).

### Output of the rating space task

**Figure 5.**
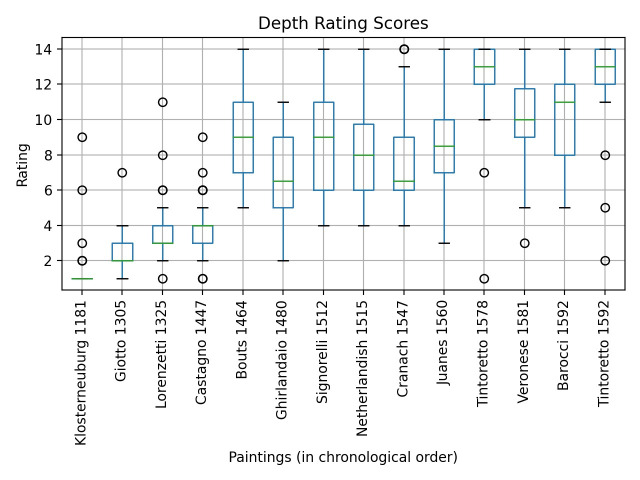
Depth rating of each painting in percentages—0% signifies the lowest amount of depth and 100%, the highest. The paintings are presented in chronological order.

In the depth rating task, participants were asked to rank fourteen reproductions of the paintings on a scale from 1 = flattest to 14 = deepest. The results can be seen in Fig. 5. It shows on the one hand that participants roughly agreed in their ratings. On the other hand there is a correlation between the depth score and the historical order of the paintings. The four oldest paintings were clearly rated as the flattest in the exact chronological order (Klosterneuburg 1181, Giotto 1305, Lorenzetti 1325, Castagno 1447). The group of paintings receiving average scores are the ones in the chronological middle of our sample (Bouts 1464, Ghirlandaio 1480, Signorelli 1512, Netherlandish 1515, Cranach 1547, Juanes 1560). The four paintings with the highest scores are the latest ones (Tintoretto 1578, Veronese 1581, Barocci 1592, Tintoretto 1592). The correlation between depth and the time of creation is not surprising: From the fourteenth to the sixteenth century the representation of space was a major challenge and the implementation of different forms of perspectives a major topic for most European painters.

## Discussion

The aim of the present study was to investigate whether and how the structure, i.e., the “composition,” of paintings affects the saccadic movements of viewers’ eyes. We expected that composition would be mirrored in frequently

repeated saccade patterns, rather than in any specific sequential progression of the gaze through the work. We registered both the eye movements of participants and their evaluation of the composition of fourteen paintings, and developed an algorithm to calculate for each painting the similarities 1) between the saccades of the different participants; 2) between compositional drawings by the different participants; and 3) between saccades and compositional drawings of the same painting.

Our first hypothesis was that participants would largely agree in their assessment of the compositions of pictures when asked to visualize them by drawing lines. We thus expected significant similarities between the compositional lines drawn during the experiment for each painting. This hypothesis was confirmed: for all paintings the lines of composition drawn by different participants were significantly more similar to each other than to lines drawn for any other painting. Remarkably, the amount of similarity varied much between the paintings. We found the highest level of agreement in the compositional analysis of Tintoretto 1592 (Fig. A13), Castagno (Fig. A4), Giotto (Fig A2) and Ghirlandaio (Fig. A6), and, to a lesser degree, for Tintoretto 1578 (Fig. A11) and Cranach (Fig. A9). From an art historical point of view those are the pictures having the clearest, hence most explicit composition. Castagno and Ghirlandaio used a classic composition for painting the Last Supper, a model known for centuries and, for example, also used some years after Ghirlandaio by Leonardo in his renowned fresco in the refectory of Santa Maria delle Grazie in Milan. What is typical of this model is that Christ sits in the symmetric middle of his apostles at the back of an elongated table, parallel to the picture plane. Only Judas, the traitor, has a seat on “our side” of the table. All of the faces are placed along one horizontal line at regular intervals. Giotto's painting, with figures distributed on three sides of a table, is less classical but just as simple; most of the heads are placed at equal intervals on two horizontal lines (art historians describe this as “isocephalism”). Cranach’s composition is also rather straightforward, with apostles sitting at a round table seen from above, and all their heads placed along an oval line. For Tintoretto, the case is different. He deliberately deviated from the classical canon of Last Supper depictions, introduced asymmetries, added new elements, and created more multipart, complex compositions. Nevertheless, he also used clear construction lines—diagonals, horizontals and some verticals—both when rendering the architectural space and placing figures, especially their faces, within this space. Some of those lines coincide with the construction of his innovative perspective with a focal point shifted from the traditional center to the left (in the 1578 painting) or right (in the 1592 painting). The fact that test subjects agreed to a large extent on Tintoretto's compositional lines shows that these lines are quite clear—at least for students of art history. The compositional analyses of Lorenzetti, Juanes, Bouts, and Veronese have the lowest similarity scores. From an art historical point of view these pictures do not have such a clear composition as those just discussed. Salient elements—such as faces, hands and architectural features—are not, or at least not as clearly, distributed along specific lines. The enamel from Klosterneuburg, Signorelli, Barocci and the anonymous Netherlandish painter achieve intermediate values. Thus, our results confirm the first hypothesis and lead to an interesting though rather logical conclusion: The clearer the composition, the greater the agreement in the assessment of it.

Our second hypothesis was that there would be similarity between the saccades of participants viewing the same painting. This too was confirmed: For all of paintings the saccades of different participants were significantly more similar to each other than to the saccades made while viewing any other painting. Here again however, the scores vary from painting to painting. Castagno (Fig. A4), Giotto (Fig. A2) and Ghirlandaio (Fig A6) achieve the highest similarity scores; Barocci (Fig. A14), Tintoretto 1592 (Fig. A13) and Veronese (Fig. A12) the lowest. It is remarkable that the similarity of saccades within participants correlates highly with the similarity between participants. We can therefore conclude that the similarity of saccades depends more on the stimulus and less on differences between individuals. Since the pictures with the lowest similarity scores are also the ones that appear to have the highest visual complexity, i.e., a higher amount and greater diversity of painted elements, we assume that the variety of saccades increases with the visual complexity of a painting. (For a discussion of how to assess the complexity of pictures cf. Commare et al., 4).

Our third hypothesis assumed a significant similarity between compositional lines, as evaluated by participants, and the saccades they performed while viewing the same painting. This hypothesis was confirmed, though only for twelve of the fourteen paintings in our study—Barocci and Bouts do not conform (and Juanes was not significant in this instance). The hypothesis was also supported by the fact that the similarity between saccades and composition was higher on an individual level (within participants vs. between participants), at least for most of the paintings (Fig. 4B). Castagno, Ghirlandaio and Giotto had the highest similarity scores between compositional drawings and saccades. As discussed above, these are the most explicit and classical compositions in our sample. In all three paintings a great number of saccades followed the horizontal axis, and some the vertical, as they were detected by the participants as the main lines of composition (axes connecting the torso and faces of the apostles, their feet, but also architectural elements).

Although the third hypothesis was generally confirmed, the cases contradicting it deserve a closer inspection. How can we explain the deviations between saccades and composition lines that occurred for two out of fourteen paintings? To what extent do we need to adjust the hypothesis? Firstly, there is a general explanation: Our results show that the similarity between saccades and compositional drawings correlates 1) with the similarity of saccades, and 2) with the similarity of compositional drawings (Fig. 4A). The obvious interpretation is therefore that paintings with lesser explicit compositions trigger a higher diversity among the lines drawn by the participants as well as a higher diversity among their saccades. Since for such paintings both the compositional lines and the saccades are more varied, their comparison leads to higher diversity scores. Bout’s Last Supper (Fig. A5) is a good example of this. The compositional lines drawn to analyze it (Fig. A5) are less consistent than, for instance, those drawn for Barocci’s painting (Fig A14). This is evident on sight and is confirmed by the algorithmic analysis (Fig. 3B). Secondly, the visualization of data collected for Barocci’s Last Supper show clear differences between the compositional axes and the most frequent saccades. Most of the lines drawn by participants analyzing this painting (Fig. A14) fall among the following groups. 1) The two diagonals of the picture, crossing each other in the center. They are, on the one hand, the diagonals of the rectangular plane of the picture, and on the other hand, spatial lines leading from the angels (top) and servants (bottom) in the foremost plane at the corners of the picture, into its depth—that is, to Christ’s head. The diagonals serve as though they were part of a central perspectival construction, although, in the proper sense of the word, they are not: the vanishing point of this painting is on the mid-perpendicular of the painting, clearly above Christ’s head. 2) Horizontal lines, especially those running along the height of the table and the heads of the apostles. 3) Some vertical lines that refer mainly to the spatial architecture depicted in the work, especially along the central axis. 4) Some curves that, just like the diagonals, mostly point to similarities between the symmetrical design of the surface of the painting and its represented space. Those composition lines roughly meet the art historical expectations. If we turn to the visualization of the saccades of the participants viewing this painting (Fig. A14), it is evident that they also repeat specific patterns, but patterns that are different from those of the compositional drawings (aside from a couple of drawn horizontal lines). For instance, as the head of Christ was looked at very often, lot of saccades converge there. But hardly any of them correspond to the drawn diagonals. It seems that the fixations of the viewers are focused on specific spots of the painting—heads, hands in action, the dog. Most saccades connect these fixations, usually along the shortest route. The saccades of participants viewing Bout’s Last Supper feature a similar structure (Fig. A5). They also connect centers of interest, often heads—of Christ and his apostles, as well as the portraits in the background. Many viewers were also interested in the houses that can be seen through the windows on the left, a detail that was not captured by any compositional drawing. We will return to those differences between compositional drawings and saccades in the “Limitations” section below.

In our fourth and last hypothesis, we assumed that the representation of space influences both the drawn compositions and in a similar way the saccades of the participants. First of all we found that participants did agree when rating the amount of depth represented, but only roughly. When comparing the superimposed compositional drawings and saccade visualizations, it seems that spatial representation influences our understanding of composition, but does not significantly affect our saccades. Thus our hypothesis was only half true. The analysis of Barocci's picture shows the difference in relevance of spatial representation for composition lines vs. that for saccades. This difference is even more clear when looking at both paintings by Tintoretto (1578 and 1592), where he uses space in a highly innovative manner. As discussed above for the later painting, most participants underline in their drawings the diagonal structure of the perspectival space (Figs. A11 and A13). In contrast, the saccades of the same participants run more horizontally than along the space-defining diagonals, where they are only partial (Figs. A11 and A13).

Our study thus provides strong evidence for the traditional assumption in art literature that pictorial composition guides the movement of the eye. We thus contradict the results of Garbutt and Spehar (7) and Kirtley (11) who denied such correlations. However, this is not a real contradiction. Although their studies were based on the same question, they focused on the sequential gaze path of participants, whereas our study analyzed the cumulative saccades of participants taking time (60 sec) to view each painting. Our data shows 1) that different participants repeat similar eye movements; 2) that saccades form specific patterns just like their compositional analyses; and 3) that there is a significant correlation between the patterns of the saccades and the patterns of the compositional analyses. This correlation 1) is higher on an individual than on a collective level; and 2) varies greatly from picture to picture. We found the highest similarity for paintings that organize the characters and main objects of the Last Supper along simple horizontal and/or vertical lines on the surface of the painting (as in Figs. A2, A4, A6).

### Limitations

While the present study delivers innovative methods and interesting findings for research into the perception of pictorial composition, it does possess several limitations. Some of them are on the technical side: Firstly, the small sample of stimuli (fourteen) and their similarity limits both the statistical validity and the generalizability of the study. Future studies might apply our methodological tools to test a larger amount of more diverse pictures. Secondly, that the group of participants were art history students might bias the results. Future studies might experiment with the effect of art expertise in the perception of pictorial composition, as well as the possible effects of gender, since all of our participants were female. A third and more fundamental limitation is our loose definition of the pivotal term “composition.” Both in the conceptualization of the study and in the execution of the experiment we opted for a generic definition of this term, one that is often used in the field of art history. However, we are aware that this could confound the results, as a “compositional line” might connect elements in a picture on a formal and/or content level in a two-dimensional painting surface, or a three-dimensional perspective (of represented space); thus, as long as a participant does not explain the lines she has drawn, it is often not possible to exactly understand what she intended to underline. In a future study it might be advisable either to work with a more precise definition and make it clear to the participants, or to ask them to explain what they intended to denote with their drawn lines. A more precise definition should also be the starting point for clarifying commonalities and/or differences between pictorial composition on the one hand and the saccadic connection of nearby salient elements on the other.

## Ethics and Conflict of Interest

The authors declares that the contents of the article are in agreement with the ethics described in http://biblio.unibe.ch/portale/elibrary/BOP/jemr/ethics.html and that there is no conflict of interest regarding the publication of this paper.

## Acknowledgements

We wish to thank Kristina Miklosova and Joanna Chaffin for their assistance with data collection. We thank Martin Warnke (Lüneburg) who indirectly stimulated this study by persistent questioning.
